# 

**Published:** 1873-02

**Authors:** 


### EDITORIAL, ETC.

---

*The Thirty-third Annual Commencement of the Baltimore College of Dental Surgery* will be held at Concordia Opera House, on Thursday evening February 27th, 1873.

The "Valedictory Address" will be delivered by W. W. H. Thackston, M. D., D. D. S., of Farmville, Virginia, a graduate of the class of 1842, and the only survivor of this, the second class of the Baltimore College.

The "Class Address" will be delivered by Jerome B. Ten Eyck, M. D., of Michigan, a member of the graduating class. The graduating class will be one of the largest ever sent forth by the Baltimore College of Dental Surgery, and will contain the first female member.

The Alumni and friends of the Baltimore College of Dental Surgery are cordially invited to be present at the annual commencement exercises, without further notice, as it is impossible for the Dean to find time at the close of the session to send special invitations to all.

